# Significance of Whole-Genome Sequencing for the Traceability of Foodborne Pathogens: During the Processing of Meat and Dairy Products

**DOI:** 10.3390/foods14081410

**Published:** 2025-04-18

**Authors:** Kai Dong, Danliangmin Song, Shihang Li, Xu Wang, Lina Dai, Xiaoyan Pei, Xinyan Yang, Yujun Jiang

**Affiliations:** 1 Key Laboratory of Dairy Science, Department of Food Science, Ministry of Education, Northeast Agricultural University, Harbin 150030, China; dk283070004@126.com (K.D.); cssdlm19961022@vip.qq.com (D.S.); 354575268@qq.com (S.L.); 2 Center for Dairy Safety and Quality, National Center of Technology Innovation for Dairy, Hohhot 010110, China; wangxu35@yili.com (X.W.); lydailina@yili.com (L.D.); peixiaoyan@yili.com (X.P.); 3 Food Laboratory of Zhongyuan, Luohe 462300, China

**Keywords:** foodborne pathogens, WGS, traceability, food industry, typing

## Abstract

The complexity of tracing foodborne pathogens in the food chain has increased significantly due to the long and complicated chain, the involvement of numerous links, and the presence of various types of pathogens at different stages and environments. Traditional typing techniques are not sufficient to meet the requirements of tracing pathogens in the food chain. Whole-Genome Sequencing (WGS) has gradually become an important technological tool for characterizing and tracing pathogens in the food chain due to comprehensive information, speed, and superior discriminatory power. This paper provides an overview of the advantages of WGS and its application in foodborne pathogen traceability. This paper focused on foodborne pathogen contamination pathways during the processing of animal foods in commercial restaurant kitchens and the potential contamination of milk, milk powder, and other dairy products by pathogens during processing in the dairy industry chain and environments. Improper handling practices during meat processing (i.e., using cloths, washing hands without soap, and cleaning boards with knives) were a critical point of foodborne pathogen cross-contamination in commercial kitchen premises. However, in dairy products, contamination of pathogens in raw milk was the main cause of foodborne disease outbreaks. Therefore, preventing the contamination of pathogens in food should not only be focused on hygiene measures during processing and in environments but also on the quality and hygiene of raw materials to prevent the spread of foodborne pathogens throughout the entire production chain. Further, Whole-Metagenome Sequencing and DNA sequence markers are considered to be the future direction of WGS. The purpose of this work is to promote the wider application of WGS during the processing of meat and dairy products and provide theoretical support for the rapid investigation and accurate traceability of foodborne pathogen outbreaks in food.

## 1. Introduction

One of the most crucial assurances of good health is safe food. According to the World Health Organization (WHO) [1], as many as 600 million, or almost 1 in 10 people in the world each year, fall ill after consuming contaminated food. In addition to having a significant negative influence on people’s health, foodborne disease also has serious economic repercussions for families, societies, businesses, and nations [2]. However, as people’s living standards continue to improve, their demand for diversified and high-quality food also increases, which has led to an increasingly complex supply chain in the global food industry [3]. However, this complexity has increased the risk of outbreaks of foodborne diseases, making it much more difficult to trace their origins [4]. Therefore, the most important issue currently is how to quickly and accurately identify the sources and transmission pathways of foodborne pathogens, including determining how pathogens are introduced during food production and processing [5].

In recent years, organic and free-range foods have gradually become more and more popular and favored by more and more people because they are more natural, healthy, and nutritious, which meets the dietary needs of modern people for health. As a result, the production and processing of animal products occur not only in slaughterhouses and various facilities but also increasingly in commercial restaurant kitchens, which have become important stages in this process. Interestingly (and quite the opposite), China’s dairy industry has moved toward scale and industrialization, with the growing demand for dairy products and the increase in dairy processing technology. The developments of dairy industrialization and modifications of animal food production techniques have increased the spread of foodborne pathogens [6]. Due to the relative complexity of its industrial chain, long chain, and different types of foodborne pathogens in different links, their production and evolution patterns are different, so the difficulty of traceability has increased dramatically [7].

When pathogens occur in the food industry chain, modern microbial molecular typing techniques can be used to compare the homology between contaminated pathogens and those contaminated in different segments and pathways to trace the origin of the pathogenic organisms. This process requires rapid, accurate, and complete typing techniques. Traditional pathogen typing techniques have been eliminated due to time constraints and an inability to accurately identify pathogens [8]. Molecular typing techniques have been widely used for the traceability and analysis of foodborne pathogens because of their speed and accuracy, but they still have disadvantages such as low resolution and discomfort for large-scale operations. With the development of sequencing technology and bioinformatics algorithms in the past ten years, WGS technology has emerged as an excellent technology for characterization and traceability, due to comprehensive information, speed, and superior discriminatory power [9].

At present, WGS is generally used for outbreak investigation, source identification, and prevention monitoring of foodborne pathogens [10]. Although the use of WGS technology in the traceability of foodborne pathogens has been summarized in some literature [11,12,13,14,15,16], there was still a lack of summary and analysis of its application in food processing, particularly during the processing of meat and dairy products. Therefore, this paper briefly summarizes the development process of foodborne pathogen detection technology, and explains the advantages of WGS in foodborne pathogenic traceability. We mainly focused on foodborne pathogen contamination pathways during the processing of meat products in domestic kitchens and the potential contamination of milk, milk powder, and other dairy products by pathogens during processing in the dairy industry chain. This paper also analyzes and summarizes the current issues and future development directions of WGS technology. The purpose of this paper is to promote the use of WGS in the meat and dairy industry chain and provide theoretical support for the quick detection and accurate traceability of foodborne pathogens.

## 2. Typing Traceability Technology

Various bacterial typing techniques have advanced significantly in recent years [17]. Traditional typing and tracing methods have been constantly being refined and enhanced and have played a crucial part in the identification of outbreaks of pathogenic bacteria [8,18]. Due to its exceptional discriminatory power, WGS has garnered significant attention for its application in genotyping and traceability [19]. Wei et al. [8] described in detail the typing and identification methods of foodborne pathogens, so we will not go into too much detail here. We briefly generalize the advantages and disadvantages of common foodborne pathogen typing traceability technology, shown in Table 1.

### 2.1. Traditional Technology

#### 2.1.1. Biochemical Typing

Biochemical typing was based on the biochemical properties of pathogenic bacteria, including the morphological and physiological characteristics of microorganisms. Biochemical typing mostly includes serotyping methods, antibiotic sensitivity typing, and phage typing [8]. The primary drawback of these phenotypic approaches was that some of the collected traits may alter depending on the stage of bacterial development or the effect of external stimuli. As a result, the method has significant drawbacks and cannot be used to detect emerging foodborne diseases [20]. Traditional typing techniques cannot provide enough information to distinguish between virulent strains when tracking the source of an illness.

#### 2.1.2. Molecular Typing Techniques

The use of molecular typing techniques for traceability identification and epidemiological analysis of foodborne pathogens has increased recently due to their advantages of high speed, high accuracy, strong operability, and high typing rates [21]. As molecular typing can reveal that pathogenic bacteria isolated from cases, food manufacturing staff, or food environments might come from a common source, it was invaluable for tracing and tracking pathogens along the food chain [22].

One of the most crucial technologies for analyzing the genetic variety of bacterial strains was pulsed-field gel electrophoresis (PFGE) typing, which was favored by scientists for its high resolution [23]. When it comes to strain typing in bacterial illness outbreaks, it is regarded as the “gold standard” [24]. The CDC’s PulseNet program used PFGE data for molecular surveillance. By collecting and analyzing laboratory data, they compared enteric bacteria to identify clusters of food and waterborne disease that might otherwise go unrecognized. But the PulseNet program has been transitioning to WGS since 2013, and PFGE has been completely discontinued in spring 2019 [25]. The drawbacks of PFGE are mainly that the results of the electrophoresis can be easily influenced by human variables, and the process was difficult and time-consuming to execute. As a result, it was necessary to have highly skilled laboratory personnel and was unsuitable for epidemiological studies involving a large number of isolates.

PCR-based DNA molecular marker technologies include restriction fragment length polymorphism (RFLP) [26], randomly amplified polymorphic DNA (RAPD) [27], and amplified fragment length polymorphism (AFLP) [28]. RFLP was the first molecular labeling technique employed, but it was difficult to apply in experimental settings due to its lengthy detection duration, high cost, and inconvenient operation on a wide scale [29]. The benefits of RAPD are its quick operation, minimal experimental cost, and reasonably good resolution. But RAPD has some issues with the consistency and reliability of results [30]. AFLP combines the benefits of RFLP and RAPD. AFLP had the advantages of high sensitivity, good precision, good repeatability and high information content [31]. The high requirements for personnel, high quality of formwork, and the hefty cost limited its adoption.

**Table 1:** Advantages and disadvantages of some foodborne pathogen typing traceability technologies.

Methods			Advantages	Drawbacks	References
Traditional pathogen typing		Serotyping	Simple Fast Good repeatability	Difficult to find new antibody antigens Lower resolution No effective identification the pathogens	[31]
		Antibiotic sensitivity typing	Strain typing and evolutionary studies can be performed based on multi-drug resistance characteristics	The resistance genes can change Different strains may have same resistance map	[31]
		Phage typing	Phage-lysing bacterial cells exhibit host specificity	Phage type incomplete Phage typing may have multiple results	[32]
Genotyping methods	Based on enzyme digestion technology	PFGE	High accuracy Good repeatability High typing rate The ‘gold standard’ for bacterial typing	Complex operation Take a long time Low sensitivity and specificity Affected by manmade mistakes	[33]
	Based on PCR technology	RFLP	Good repeatability High stability High specificity	Cumbersome experimental operation Long testing period High cost Not convenient for large-scale operation	[25]
		AFLP	High sensitivity Good accuracy Good repeatability High information content	High requirements for personnel High quality of formwork Expensive	[27]
	Based on sequencing technology	MLVA	High sensitivity Good repeatability Simple and efficient	Certain requirements for tandem repeat sequences High operational requirements for personnel screening sequences The sequences screened can affect the results	[34]
		MLST	Great typing ability Good repeatability Storable results Easy data sharing	High sequencing costs Genes may be mutated in conserved sequences	[35]
		WGS	Greater discriminatory Complete information Analyze the source of foodborne pathogens Surveillance of foodborne pathogens	Large amount of data Lack of correct interpretation High cost of use and maintenance	[12]

Note: PCR (polymerase chain reaction), PFGE (pulsed-field gel electrophoresis), RFLP (restriction fragment length polymorphism), AFLP (amplified fragment length polymorphism), MLVA (multiple locus variable-number tandem repeat analysis), MLST (multilocus sequence typing), and WGS (Whole-Genome Sequencing).

Multilocus sequence typing (MLST) [36] is a technique to amplify highly conserved sequences in strain genes and compare their allelic variations for typing traceability. Allele and sequence-type data of the strains can be searched and compared online through the MLST database of the corresponding species (https://pubmlst.org). MLST has been used for epidemiologic surveillance and evolutionary studies of a wide range of bacteria due to its simplicity and ease of comparison between different laboratories and has become a routine method of typing bacteria. High sequencing costs and the inability to fully type bacteria limit the applicability of MLST (only up to seven housekeeping genes can be typed), despite its great typing ability, good reproducibility, storable results, and ease of data sharing.

### 2.2. WGS Technology

#### 2.2.1. The Development of Gene Sequencing Technology

Sequencing technology has developed from the first-generation Sanger technology in 1977 [37] to the present day, spanning over 40 years. In the course of this technological evolution, sequencing read lengths have fluctuated from long to short and back to long. Although the first-generation sequencing technology had high accuracy, its complex operation process, high cost, and low throughput hindered its widespread application. Subsequently, in 2005, a simple and rapid high-throughput sequencing method called pyrosequencing was reported in the journal Nature, marking the beginning of the second-generation sequencing technology [38]. While the second-generation sequencing technology significantly reduced sequencing costs, and increased sequencing speed and accuracy, its sequencing read lengths were relatively short, approximately 100–150 bp. In 2011, the launch of the SMRT (single-molecule real-time) sequencing technology by PacBio marked the beginning of the third-generation sequencing technology [39]. The most significant feature of third-generation sequencing technology is single-molecule sequencing, which eliminates the need for PCR amplification, and provides ultra-long-read lengths. However, it has the drawbacks of high sequencing costs, high error rates, and random occurrence of errors. The introduction of Nanopore sequencing by the British company Oxford Nanopore Technologies signified the beginning of fourth-generation sequencing technology [40]. The characteristics of fourth-generation sequencing technology include fast sequencing speed, real-time monitoring of sequencing data, and convenient portability of machines, but the cost of single-chip sequencing still exceeds several hundred US dollars.

In 2012, Adam Phillippy and his colleagues developed a new fusion technology that combines second- and third-generation sequencing technologies to generate nearly error-free long-read sequencing technology. The emergence of long-read sequencing technology has greatly improved genome sequencing and transcriptome assembly. Whole-Genome Sequencing (WGS) technology primarily relies on the combination of second- and third-generation sequencing technologies and has seen new developments based on the foundation of second-generation sequencing technology. Currently, in genomic research, WGS allows for a detailed comparison of similarity among strains of foodborne pathogens, and it can be used for the assessment of strain relatedness based on inferred phylogenetic relationships, evolution, and functional capacity. Therefore, it has become an invaluable tool in foodborne pathogen surveillance, characterization, and source tracking.

#### 2.2.2. The Development and Application of WGS

**Figure 1:**
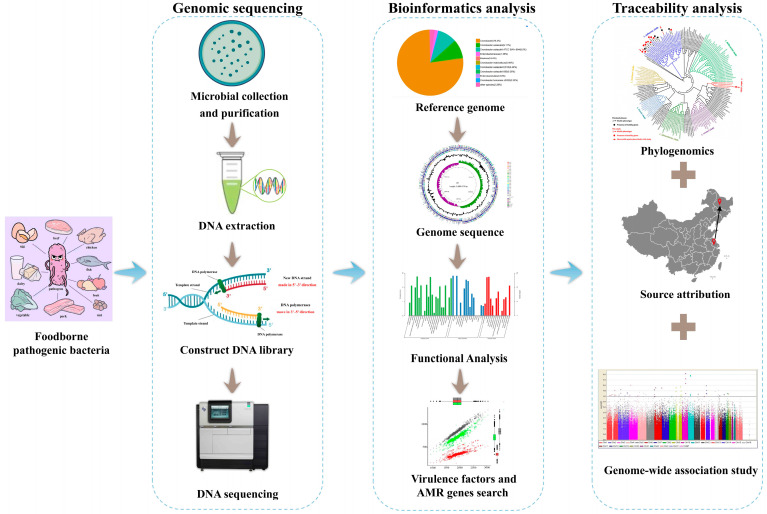
Common workflow of a WGS study.

We have entered the genomic era as a result of rapid evolution of next-generation sequencing (NGS) technology in recent years [41,42]. WGS is a powerful technology that can sequence the entire genome of pathogenic bacteria, providing a wealth of information for analyzing virulence and drug resistance [43]. WGS usually consists of several steps: obtaining pure cultures of the organism of interest, DNA extraction, constructing DNA libraries, and DNA sequencing, followed by possible comparisons, data analysis, and finally biological interpretation [12]—the common workflow is described in Figure 1, and the currently commonly used tools for bioinformatic analysis are shown in Table 2.

The integrity of Whole-Genome Sequencing (WGS) data allows it to circumvent the classification limitations brought about by only examining a small portion of the genome, making it an increasingly effective technological tool for precise analysis and tracing back origins [44]. Whole-Genome Sequencing (WGS) offers a significantly higher discrimination resolution compared to conventional subtyping methods, amplifying it by hundreds to thousands of times. Consequently, WGS-based subtyping presents notable advantages in enhancing microbial food safety surveillance through improved discriminatory capabilities. By comparing the Simpson index of the two typing techniques, Stasiewicz et al. [45]. found that the discriminatory power of PFGE was significantly surpassed by WGS-based typing.

WGS is a technology that allows for the sequencing of the entire genome of a pathogen, providing complete genetic information. In outbreak investigations, when combined with One Health approach (an integrated approach emphasizing the interconnectedness of human, animal, and environmental health), WGS enables researchers to analyze pathogens not only from human cases but also from animals and the environment. This comprehensive perspective aids in understanding the pathways of disease transmission and their origins. Furthermore, WGS can be effectively integrated into food safety and public health inspection processes, assisting in the detection and identification of various issues related to outbreaks, such as pathogen introduction, concealment, cross-contamination, source attribution, and temporal and geographic distribution.

Moreover, the accessibility of WGS data online enables the rapid application of WGS-based subtyping for swift responses to cross-contamination outbreaks occurring in diverse geographical locations. WGS technology enhances the ability to track cross-border epidemics, effectively identifying and analyzing foodborne pathogens across different countries. Thepault et al. [46] used a pan-genomic set of 1810 genes to evaluate and discover 15 novel host-specific genetic markers that allowed clinical isolates from France and the UK to be linked to ruminants and chickens, although potential geographic variations between these sources. WGS technology can analyze the microbial sequences of an epidemic, and by examining the food supply chain during the tracking and tracing process, it can determine whether they are related to the same transmission chain [47].

Due to its outstanding application value and development potential in the routine monitoring and risk assessment of foodborne pathogens, WGS has attracted widespread attention from food regulatory agencies and public health scientists [48]. They use WGS to monitor sequence databases and identify indistinguishable isolates from patients, the food chain, and cluster clinical isolates, thereby providing early warning of the risk of foodborne disease outbreaks. After identifying potential associations, food regulatory agencies investigate the traceability of the implicated foods, confirm the link between the outbreak and the food, and identify the root causes of the outbreak to implement effective control measures [49]. Through the analysis of case studies and the integration of epidemiological investigation results, support is provided for potential food sources of pathogens. Utilizing WGS for routine public health surveillance of regional foodborne pathogens can uncover more clustered outbreaks, even very small ones (e.g., just two clinical cases), by comparing the genome sequences of patients with those already sequenced from food samples or other cases. This high-sensitivity detection capability surpasses what traditional typing methods can achieve [47].

WGS can be used to evaluate contamination in the food business in addition to routinely monitoring foodborne pathogens. After an outbreak of foodborne illness, if a pathogen is found in a place where food is produced or consumed, its genetic sequence could be compared to a database of human pathogens [50]. WGS indicated that foodborne pathogenic bacteria have contaminated the food if the genetic sequences were similar. Nevertheless, even if no human disease was detected, the presence of pathogens in food or essential food processing environments indicated a breach in sanitation and prompted a regulatory agency investigation [22].

WGS has become the preferred tool for outbreak investigations. COVID-19, caused by a novel coronavirus infection that swept the world in 2019, posed a serious threat to human health [51]. It has become a global priority to overcome the new coronavirus. Since then, researchers used the WGS to obtain the genome sequence information of the new unknown virus for the first time. WGS can provide further confirmation for patients who exhibit highly suspected clinical phenotypes of the new coronavirus, but have tested negative on RT-PCR nucleic acid tests. The gene sequences of all potentially pathogenic microorganisms, including the novel coronavirus, can be identified by WGS and provide a valuable reference for understanding pathogenic information related to multiple or secondary infections. WGS has uncovered many secrets behind COVID-19, such as the origin and the mechanism of COVID-19 infection and pathogenesis, which has helped in the prevention and control of outbreaks and subsequent research [52].

## 3. Application of WGS in the Traceability of Foodborne Pathogens

Early detection and identification of foodborne disease outbreak causes can minimize harm [46], but the complexity of the farm-to-table process increases traceability challenges due to the lengthy food chain and numerous links.

### 3.1. Application of WGS in the Meat Industry Chain

Natural and organic foods have seen a spike in popularity among consumers in recent years. This has caused the preference for “natural” meat (meat products grown in natural environments without the use of pesticides, fertilizers, growth hormones, preservatives, or other chemical additives during the growing and processing stages) and meat products and the rising popularity of organic and free-range farms. The rise in the production and consumption of meat has resulted in increased concerns regarding the safety of these products for consumers. While bacterial contamination of raw meat products occurs, the consensus is that most meat-related human illnesses arise as a result of either consumer or retail outlet mishandling of the raw products (cross-contamination to other foods), improper cooking, or improper storage of products after preparation.

**Figure 2:**
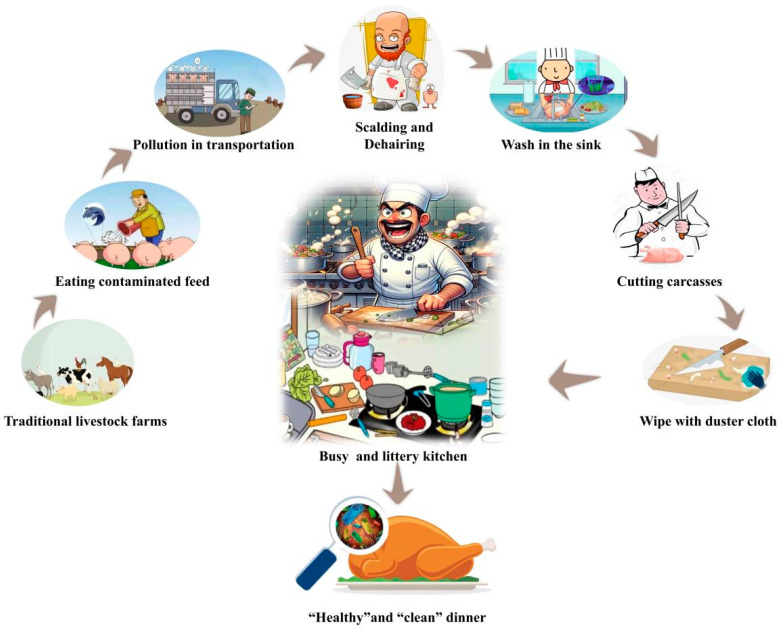
The transmission path of foodborne pathogens in the meat product industry chain.

However, are these consensuses necessarily entirely correct? No, we believe they are one sided. Wang et al. [53] found that pathogens found in the gastrointestinal tract of livestock may also transfer to the exterior of meat products through feces or during processing (Figure 2), Salaheen et al. [54] and Doyle et al. [55] also found this phenomenon. Therefore, how can the harm of foodborne pathogens be minimized to the greatest extent? To achieve this, it is necessary to understand the transmission routes of foodborne pathogens. As a result of the complex nature of the production and processing environments (farm to fork), pathogen contamination can occur at any level (Table 3). Consequently, the complexity of meat production/processing systems presents a significant challenge in reducing contamination of pathogens in the final products.

Some common serotypes of foodborne pathogens exhibit high clonality, and traditional typing techniques lack sufficient discriminatory power to determine the genetic relatedness of strains, which cannot meet the detection needs [74]. Whilst previous molecular subtyping methods detected sequence changes in a small portion of the microbial genome, WGS captures them across the entire genome and thus more accurately describes the genetic relatedness of strains. Compared with MLST and PFGE, the large amount of information available through WGS greatly enhances our ability to determine the source of infection. In tracking and tracing, the relatedness of bacterial sequences from outbreaks as well as the food production chain is assessed to determine if they could be part of the same transmission chain. Kang et al. [9] conducted an epidemiological investigation of *Salmonella* in the duck production chain using WGS technology. The results showed that the isolation rate of *Salmonella* during the hatching stage was 25.7%, while on the farms, it was 23.6%. The highest isolation rate was found in supermarket duck meat samples, at 42.5%, which may be due to cross-contamination of *Salmonella* during production, storage, transportation, and sale processes. *S. typhimurium* isolates from various points within the duck production chain exhibited clustering, indicating potential transmission of the pathogen along the production continuum, potentially reaching the market and posing a risk to human health. Given the geographic dispersion of the hatchery, farm, slaughterhouse, and market, *S. typhimurium* may disseminate across different regions within the production chain. Therefore, the prevention of pathogen contamination in food products should focus on not only the downstream market but also the control strategies at the upstream stages, including farms and slaughterhouses, to stop the spread of pathogens along the production chain. The control of pathogens in the whole meat production chain effectively improves the food safety of final retail products and decreases the risk of transmission to humans.

In addition, handling raw meat during processing or storage is a major route of infection with foodborne pathogens. Mylius et al. [75] also found that foodborne pathogens could spread in kitchen environments, with the highest risks associated with direct contact with utensils/surfaces used for raw meat, such as knives, hands, cutting boards, faucet handles, and sponges. Lai et al. [76] used WGS technology and discovered 18 *Campylobacter* and 3 *Escherichia coli* in six samples (out of a total of ten samples). These pathogens were also found on the hands of cleaning area staff. Additionally, they discovered that one of the sequence type strains found in raw chicken—ST693—was also detected on the sink and floor surfaces. Therefore, they suggest that there may be a risk of cross-contamination between raw chicken and kitchen sinks. Additionally, *Campylobacter* was found on the floors and countertops in the cleaning and cutting areas, likely as a result of droplets containing Campylobacter. Due to consumers’ improper handling of raw meat and lack of awareness of factors affecting the safety of meat products, the likelihood of contamination with foodborne pathogens also increases.

Furthermore, ineffective cleaning and preventive measures can amplify the risks associated with unsafe practices. Habib et al. [77] found through a survey of New Zealand consumers that approximately 28.8% of respondents indicated they would not consider using a separate cutting board to prepare chicken. Additionally, in European kitchens, kitchen sponges are commonly used to clean countertops (such as cutting boards) and to wash and wipe pots, pans, and other utensils. In surveys conducted in China, dusters are often used for wiping and cleaning. However, they also found that *Campylobacter* strains isolated from cutting boards and cloths had the same genotype as those isolated from raw meat samples, indicating that contaminated surfaces act as vectors for spreading this pathogen. Cardoso et al. [78] also discovered in their study that *Campylobacter* was found on kitchen cutting boards at different contamination levels (2.2 × 10^3^ CFU/g and 4.0 × 10^1^ CFU/g) during chicken cutting. This suggests that even with low levels of contamination, improperly handled chicken may lead to *Campylobacter* contamination, spreading across the entire kitchen surface. After handling raw meat, cleaning may not be as effective as consumers expect it to be, an aspect that becomes difficult to assess given microorganisms leave no visible traces of dirt to spot.

Although the transfer of foodborne pathogens from livestock farming and slaughter to the production and processing of meat products has been well studied and acknowledged [79], in practical situations, people still tend to be careless when handling and processing livestock that has been contaminated by pathogens. The invisibility of microorganisms makes it easy for everyone to be deceived [78]. For instance, when handling livestock corpses, the instinct may be to put raw meat in a clean container owing to an unexpected accident. You may move several little cups or devices that have been arranged on the kitchen counter or on a cutting board to make room for the raw meat after handling it without washing your hands. Then, he considered that the cups he had touched with his hands were “clean” and did not require further washing. Everyday life was composed of a flow of activities that were not linear, neither predictable nor certain.

Furthermore, standardized rapid detection methods are also crucial measures for preventing the transmission of foodborne pathogens. However, variation among isolates from affected human cases can occur within an outbreak, and mutational changes could however happen at any point in the transmission pathway and the higher the resolution of the typing method the greater the likelihood of detecting such events [80]. WGS can detect genetic variations between pathogens strains that are unidentifiable by other molecular typing methods and reveal the evolution and transmission routes of pathogens [49]. Therefore, WGS will help elucidate the mechanisms of spread and identification of the source when the foodborne illness outbreak. Meanwhile, the high resolution of WGS allowed investigators and regulatory agencies to take action at a lower level of epidemiological evidence, a key advantage for the relatively small outbreaks typical for foodborne pathogens.

### 3.2. Application of WGS in the Dairy Chain

From infants to the elderly, milk and dairy products have been deemed one of the best foods for everyone [81]. However, dairy products’ high nutritional content also creates an ideal habitat for the growth of some foodborne pathogens that pose a threat to the general public’s health. As a result, it is crucial to stop and manage pathogen contamination during the manufacturing of dairy products.

**Figure 3:**
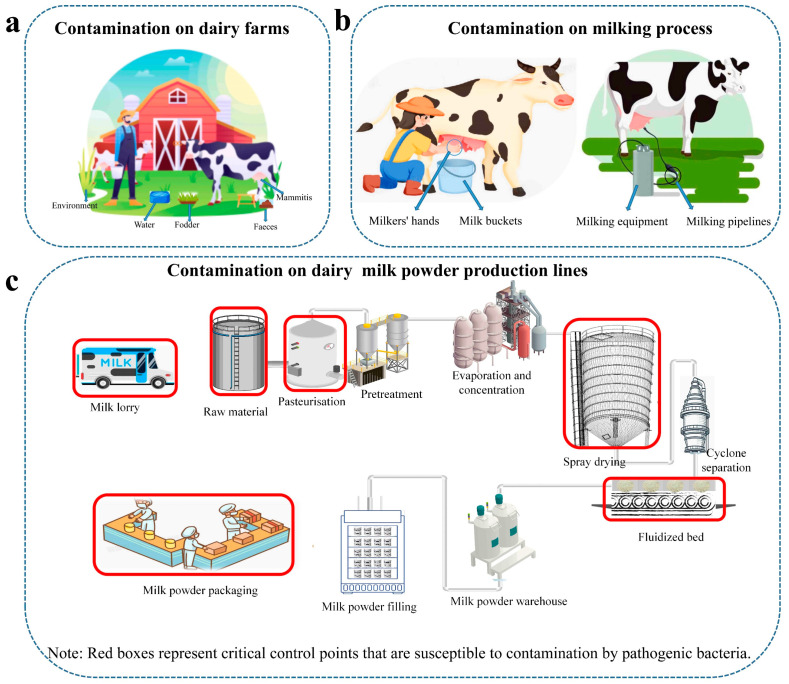
The transmission path of foodborne pathogens in the dairy industry chain, possible contamination sites on farms (**a**), during milking process (**b**) and in the production of dairy products (**c**).

Safety control of dairy products begins early before consumers consume them. The dairy farm was a dynamic habitat with a complex microbial ecosystem that is a significant host for foodborne infections. The harmful bacteria might contaminate raw milk and hence infiltrate the dairy production chain if they were present on the farm [82]. The environment of the animal farm was a crucial control point for dairy safety issues. Because of the presence of numerous pathogens on the farm, some of them might cause mastitis [83]. Bacterial colonization of the mammary glands and form a mastitis infection, then shedding during milking and contaminating the milk (Figure 3a). *Staphylococcus aureus* and *Listeria monocytogenes* were important causes of mastitis [84]. Pathogenic bacteria can be transferred to raw milk in this situation. As a result, the farm setting was regarded as a high-risk pathway for the pathogen contamination of dairy products [85]. The cleanliness of the milking procedure was also crucial, as it was considered a high-risk factor for pathogen infection.

It was crucial to prevent fecal contamination during the milking process because pathogenic bacteria (*Salmonella*, *E. coli*, etc.) were frequently found in the feces of animals like cows. To further limit contamination by harmful germs, milking equipment and pipelines must be thoroughly cleaned and maintained (Figure 3b). Tracy et al. [86] discovered *S. aureus* isolates in swabs from milkers’ hands or noses that were comparable to those identified in milk and on the outside of milk buckets, suggesting that *S. aureus* may spread between people, cows, and milk in addition. Solenne et al. [87] discovered that raw or pasteurized milk was blamed for the vast majority of dairy foodborne outbreaks. To prevent foodborne diseases, pathogen-free raw milk manufacturing was essential. Heat sterilization was the most effective method for eliminating germs, but it cannot be used in milk and dairy products because of the substantial nutritional losses that take place during this procedure.

Pasteurization was the most popular technique used to lessen the presence of pathogenic microorganisms in dairy products. To prevent further nutritional losses, the food was cooled to 10 °C right away after pasteurization. Pasteurization does not always remove all dangerous bacteria, and milk that has been pasteurized incorrectly may still contain pathogens. The CDC’s Foodborne Disease Outbreak Surveillance System data revealed an increase in the incidence of dairy disease outbreaks since 1998. Most of the contaminated dairy products and trafficked food vehicles have been linked to unpasteurized milk [88].

However, the pasteurization of raw milk does not prevent microbial contamination of milk or dairy products. The danger of harmful bacteria contamination was also increased by inappropriate sanitation practices during or after pasteurization, incorrect handling, transportation, or storage in the plant environment and equipment. The *Cronobacter* was thought to be the most polluted during the preparation of dairy products (milk powder). Although the prevalence of *Cronobacter* in dairy products has decreased recently as a result of a heightened understanding of its ecology, product contamination still happens [89] (Figure 3c).

One of the top four producers of premium milk powder in the world—Abbott, ordered an emergency recall of its goods on 18 February 2022, in response to complaints of bacterial illnesses in newborns who had been using the company’s branded formula. Between September 2021 and January 2022, four infants contracted illnesses and two of them passed away after ingesting allegedly tainted formula. The FDA revealed in March 2022 that the formula Abbott had recalled had been connected to serious *Salmonella* and *Cronobacter* illnesses in four newborns, two of whom passed away from *Cronobacter* infections. The FDA took environmental samples from the facility and tested positive for *Cronobacter* in the filling and drying areas of the milk powder [90].

Spray drying and the preceding pasteurization phase were thought to be fully effective for inactivating *Cronobacter* in the current method, despite earlier studies showing that spray drying alone was unable to completely inactivate *Cronobacter* in milk powder manufacture [84]. *Cronobacter* contamination during post-processing may also result from contaminated materials being added to milk powder or through cross-contamination of the drying area (fluidized bed) and packing area environments.

In three distinct provinces of Zambia, Phiri et al. [91] collected 1939 samples over the course of two sampling sessions from all farms, milk collection facilities, informal milk trading farms, and industrial milk processing plants. Eventually, the similar *S. aureus* isolates were discovered on other farms, proving that the strains were communicable there. The movement of cattle, wildlife, or people might be to blame for the spread of *S. aureus* strains between farms. The results indicated that *S. aureus* strains might be spread along the dairy chain and stayed in the chain for at least 3–5 months because comparable isolates were discovered in several locations during the first and second sampling periods. This could be brought about by *S. aureus* strains remaining persistent on the farm and spreading to other facilities or remaining persistent in non-farm facilities.

### 3.3. Other Applications of WGS

Foodborne disease outbreaks and scandals have brought attention to the risk of foodborne pathogens, which has led to an increase in research institutions and regulatory authorities in the use of WGS for outbreak identification and surveillance, as well as for traceability and contamination investigation based on this technology.

The first WGS-based surveillance network for foodborne pathogens, GenomeTrakr Network was created in 2013 through a collaboration between the U.S. Food and Drug Administration (FDA), the Centers for Disease Control and Prevention (CDC), and the U.S. Department of Agriculture Food Safety and Inspection Service (USDA FSIS) [92]. To quickly track the origin of outbreaks based on distinctive genomic features associated with geographic regions, the network would gather and share genomic sequence data of collected foodborne pathogen isolates and corresponding geographic data [93]. Following the implementation of WGS surveillance for foodborne pathogens in the United States, more and smaller outbreaks were identified, outbreaks were detected earlier, the sources of outbreaks were identified more frequently, and the total number of confirmed cases associated with outbreaks increased [94]. Additionally, the Canadian Food Inspection Agency (CFIA) has sequenced mono-allelic strains of *Salmonella* and *Listeria* using WGS to gain a better understanding of the factors that raise the risk of injury and better safeguard people against pathogenic bacteria [95].

The WGS platform not only serves as a detection and typing tool but also works as a tracing tool for epidemiological studies. The implementation of WGS in outbreak investigations has greatly improved the investigation of multi-state and international outbreaks, as the geographical signal of the pathogen helps trace the outbreak strain back to its source of contamination [92,93,96]. In December 2013, four cases of listeriosis with indistinguishable PFGE patterns were identified in one US state. Due to little exposure data at the time, no typical food was identified. In a multi-state outbreak in August 2015, 20 further cases of listeriosis were found, and the patients had isolates with five different PFGE patterns. The investigation people used WGS to trace the source of the outbreak to a cheese producer, and the source of the unsolved cases from the 2013 inquiry was also connected to the same cheese supplier. Sequencing from the retrospective sequencing showed that this producer’s cheese was the cause of further unresolved cases that dated back to 2010 [94]. WGS made it possible for investigators to connect incidents that occurred over extended periods, enabling the identification of low-level product contamination and enhancing the effectiveness of preventive measures. The WHO was notified by the UK Health Security Agency (UKHSA) of a cluster of *S. typhimurium* monophasic strain of serotype 34 of unknown origin, on 27 March 2022, which was followed by reports from several European countries [97]. WGS was used to identify the chocolate product made by firm A in factory B in Belgium as the causal food product through a traceability study of the pandemic outbreak. The investigation of the company involved found that two monophasic strains of *S*. *typhimurium* were responsible for the outbreak.

The emergence of antibiotic-resistant bacteria has grown to be a significant threat to humanity in recent years [98]. Concern over the possibility of antibiotic resistance spreading through the food chain is on the rise [99,100]. However, conventional methods do not provide pertinent data on the existence and dissemination of antibiotic resistance genes in the food chain. Compared to conventional techniques, WGS can identify antibiotic resistance genes in further investigations to ascertain their resistance and virulence [101], thus better controlling the spread of dangerous bacteria. WGS-based AMR surveillance has been adopted in the scope of the National Antimicrobial Resistance Surveillance System [92,102]. State and municipal public health agencies, as well as colleges, were already doing WGS-based AMR surveillance in the United States. Recently, WGS analysis of *Yersinia pestis* isolates from the Brazilian pork production chain and human clinical cases by Martins et al. [103] confirmed the close genetic relationship between the two types of isolates. Antibiotic resistance-associated genes with high diversity were found in all nine isolates. We should pay attention to the potential role of antibiotic resistance transmission from pathogenic microorganisms in food to humans.

WGS was also increasingly used to analyze the potential virulence factors of a specific strain of bacteria. The Virulence Factor Database (VFDB) was established to provide the scientific community with a comprehensive database and online platform for deciphering the pathogenic mechanisms of bacteria. WGS technology has been widely used to identify potential novel or mutated pathogens in abrupt disease outbreaks and ordinary clinical practice [104]. For example, Shiga toxin-producing *E. coli* (STEC), which carries a Shiga toxin (Stx-)-related gene, can develop mutations that result in the production of Stx1a-, Stx1c-, Stx2a-, Stx2c-, Stx2d-, etc. Similar to Stx2a- and Stx2c-, the Stx2d-variant may be associated with the development of hemorrhagic colitis and hemolytic uremic syndrome, but there were not many reports of this variant’s existence in STEC strains. Research showed that swine might carry Stx1a-, Stx2e-, or Stx2d-producing *E. coli* with virulence gene profiles associated with human infections [105].

## 4. Challenges and Opportunities

Even though WGS has revolutionized the molecular typing of pathogens, there are still many scientific gaps and difficulties that must be resolved to enhance the interpretation of WGS data and make WGS widely applicable in the food industry. These issues include

(1) Analysis of the vast amount of WGS data is necessary, but there are no reasonable solutions.

The analysis of the massive amount of data produced by WGS was difficult since it frequently needed specific bioinformatics knowledge and abilities. It was necessary to create new analytic software that would make it possible for non-bioinformatics professionals to interpret and analyze WGS results (perhaps with the appropriate training) [22]. Comparing unknown isolates to a reference library of previously found barcode sequences of the organism effectively detects short DNA sequences from a standard section of the bacterial genome and uses them as a reference “barcode” to facilitate improved strain traceability. If implemented, the samples could be pooled during sequencing and each sequencing sample would be easily identified during data processing. However, finding distinct DNA markers in bacteria growing in normal vs. particular conditions is challenging.

(2) WGS methods and measurement orders need to be standardized.

Although the WGS method has become an important typing tool, it is still a long way from completely replacing traditional typing. Sequencing errors may occur because of the complexity and length of the genomic sequence. Additionally, differing error rates and sequence quality may be produced by various sequencers and sequencing technologies, leading to varying sequencing results. Internationally standardized procedures for bacterial genome data collection and analysis are lacking. To provide trustworthy and consistent data, it is crucial to standardize WGS techniques. We think that by determining if several approaches can yield the same findings or by developing a standard technique, the quality of WGS sequencing may be standardized [12].

(3) Lack of sufficient epidemiological and food traceability evidence to properly interpret WGS findings.

From a biological perspective, high sequence similarity in WGS analysis suggests that isolates had recently shared ancestors. This means that clinical, dietary, or environmental isolates that are similar in the phylogenetic tree may be epidemiologically or causally connected. WGS analysis provides strong evidence that isolates were genetically related, but this does not necessarily mean that clinical cases were contracted directly from food or from the specific site where the WGS-matched isolate was obtained. This is because there may be indirect or complex relationships at any point along the farm-to-table continuum. Therefore, it was crucial to complement and facilitate the proper interpretation of WGS data by using epidemiological and food traceability evidence [47]. Because epidemiologic and bioinformatics data were not perfectly correlated when understanding the data, we still need to take into account the background information related to the origin of the pathogenic isolates, and sometimes epidemiological and food traceability evidence needs to be collected.

(4) WGS requires the selection of isolates for culture by traditional laboratory techniques.

WGS still relied on traditional laboratory operations, such as the selection and growth of distinct isolates. This strategy restricted the use of the sequencing technology’s speed and potential because most viruses normally need 1~2 day of culture before they can be used for further WGS analysis. Some microorganisms may be unculturable or in a viable but unculturable state, and each suspect species requires a separate selection and purification process. Metagenomics techniques can solve these constraints by immediately identifying and characterizing complete microbial populations in a single food or environmental sample in a single assay without the requirement for culture [106]. Applications in food safety and foodborne diseases are only projected to grow as sequencing becomes more advanced; and may include the integration of additional histological approaches, including transcriptomics, epigenomics, and proteomics. In the near future, metagenomics can also be applied because of its ability to comprehensively analyze the genomic information of all microorganisms in a sample, thus identifying possible pathogenic bacteria and their species.

(5) The high cost of maintaining and operating WGS.

WGS-based typing has gotten more and more affordable as sequencing technology has advanced. However, significant financing is needed for the construction of core sequencing facilities and bioinformatics (including initial investment, operating costs, human resources, laboratory and related infrastructure costs). Additionally, it is impossible to overlook the daily expense of reagents and maintenance services. How to reduce the cost of using and maintaining WGS-based monitoring will be a future direction [12].

## 5. Conclusions and Prospect

WGS has been extensively utilized to build genetic developmental phylogenetic trees for various types of foodborne pathogens and to comprehend the pathogenic mechanism because of its speed, precision, and completeness, which can obtain a large amount of data in a short period. The use of WGS technology for food microbial identification and traceability, thereby locking the source of foodborne disease outbreaks, has progressively become an international research hotspot. The management of foodborne pathogens is currently being put to a rigorous test as a result of the domestication of meat production and the industrialization of dairy production. Foodborne disease outbreaks can be successfully prevented by early diagnosis of persistent foodborne pathogens in the food industry chain and prompt adoption of efficient measures to prevent contamination. As a result, there was great hope for the future of WGS in the traceability of foodborne pathogens in the food chain.

The cost of WGS monitoring and use will significantly decrease with the development of bioinformatics technology, genomic sequence databases will become more sophisticated, and WGS data will become more accurate, reliable, and internationally comparable. However, WGS will need to overcome current challenges and limitations if it is to completely replace other typing methods. Firstly, all background information about the origin of the pathogenic isolate must be taken into account when interpreting the data, and sometimes the results need to be analyzed by combining knowledge from one or more disciplines, such as biology, microbiology, and epidemiology. Secondly, we still need to use WGS technology to enhance cross-contamination studies and risk monitoring of meat and dairy products to reduce or avoid contamination of foodborne pathogens due to improper cleaning or cross-contamination. Finally, there are still some issues with the quick identification of foodborne pathogens. How to rapidly identify associated disseminated cases of food of unknown etiology and identify them as outbreaks within a short period and issue risk warnings promptly to minimize the risk of foodborne pathogens are challenging issues that we need to face in our future work.

## Figures and Tables

**Table 2:** The currently commonly used tools for bioinformatic analysis.

	Scope of Application	Website
Bowtie/ Bowtie2 (2.5.4)	A tool for efficiently comparing high-throughput sequencing data, especially for short-read-length sequencing data.	https://bowtie-bio.sourceforge.net/bowtie2/index.shtml (accessed on 16 May 2024)
BWA (MEM)	Tool for comparing sequencing data, supporting both short- and long-read length sequencing data.	https://bio-bwa.sourceforge.net/ (accessed on 28 February 2010)
SAMtools (1.2)	Tools for processing and analyzing comparison results, with the possibility of sorting, filtering, indexing, converting, etc.	https://www.htslib.org/doc/1.2/samtools.html (accessed on 15 December 2015)
GATK (3.7)	Tools for analyzing high-throughput sequencing data, including variant detection, splice variant detection, RNA-seq analysis and other features.	https://wiki.rc.usf.edu/index.php/Genome_Analysis_ToolKit_(GATK) (accessed on 13 March 2023)
Picard (3.4.0)	Toolset for processing and analyzing sequencing data, including de-duplication, sorting, and format conversion.	https://broadinstitute.github.io/picard/ (accessed on 13 April 2024)
BLAST (1.4.0)	Tools for comparing and recognizing biological sequences, widely used for sequence similarity searching and annotation.	https://blast.ncbi.nlm.nih.gov/Blast.cgi (accessed on 17 March 2025)

Note: BWA (Burrows–Wheeler Aligner), GATK (Genome Analysis Toolkit), and BLAST (Basic Local Alignment Search Tool).

**Table 3:** Possible contamination sites in meat processing identified by WGS.

Years	Region	Sample	Location	Pathogens	Positive Rate	References
2015	Nanjing, China	Pork	Open-air markets	*Listeria monocytogenes*	6.9%	[56]
2018	Andalusia, Spain	Free-range pig	Slaughterhouses	*Salmonella*	12.93%	[57]
				*Campylobacter*	17.17%	
				*L. monocytogenes*	9.37%	
2013	South Korea	Pork	Slaughterhouse, processing line retail outlet local market	*Bacillus cereus*	4.41%	[58]
				*Escherichia coli O157:H7*	ND	
				*L. monocytogenes*	5.89%	
				*Salmonella*	1.20%	
				*S. Aureus*	0.83%	
				*Y. enterocolitica*	ND	
2013	Danish	Pig	Farms	*Salmonella*	40.9%	[59]
			Slaughterhouses		7.4%	
2010	Finland	Pig	feed and litter	*L. monocytogenes*	11%	[60]
			rectal swabs		1%	
			intestinal contents		1%	
			tonsils		24%	
			pluck sets		5%	
			carcasses		1%	
			meat cuts		4%	
2013	Spain	Pig	pre-scalding (slaughter line)	*Salmonella*	36.25%	[61]
			trucks		23.21%	
			cecal contents (slaughter line)		21.25%	
			Tonsils (slaughter line)		17.50%	
			ileocecal lymph nodes		16.25%	
			lairage		14.06%	
2021	Sichuan, China	Chicken	defeathering	*Salmonella enterica*	50%	[53]
			evisceration		36.67%	
			disinfection and pre-cooling		15%	
			segmentation		6.67%	
			refrigeration		3.33%	
2018	Trinidad	Chicken	cottage poultry processors	*Salmonella*	20.5%	[62]
			supermarkets		8.3%	
2022	Trinidad and Tobago	Chicken	hatcheries	*Salmonella*	7.6%	[63]
			broiler farms		2.8%	
2024	Guangzhou, China	Retail chicken meat	live poultry	*Salmonella*	67.5%	[64]
			frozen		50%	
			chilled		43.3%	
2022	Jiangsu, China	Ducks	hatchery samples	*Salmonella*	35.7%	[9]
			market samples		29.2%	
			farm samples		23.6%	
			slaughterhouse samples		9.4%	
2019	Southern China	Duck, Fish	Integrated fishery	*Escherichia coli*	55.17%	[65]
			Slaughter house		56%	
			Market		40.32%	
2021	Australia	Egg	Cage	*Escherichia coli*	20.3%	[66]
			Barn-laid		20%	
			Free-range		19.5%	
2023	Italy	Food processing environment	Meat	*Listeria monocytogenes*	17.57%	[67]
			Dairy		4.47%	
			Fish product		0.96%	
			RTE		1.6%	
			Breeding farms		0.32%	
			Large retail		0.16%	
2022	Japan	Raw food products for retailing	Feather	*Staphylococcus aureus* complex	52.63%	[68]
			Feces		12.07%	
			Chiller water		9.8%	
			Slaughterhouse environment		17.19%	
			Carcass		64.04%	
2024	Mexico	Raw chicken	fresh markets	*Salmonella enterica*	23.56%	[69]
			supermarkets		28.64%	
			butcher shops		29.68%	
2022	North Carolina, USA	Sheep	Feces	*Escherichia coli*	27.3%	[70]
			Cecal contents		21.9%	
			Carcass swab		10.2%	
			Abattoir resting area feces		20.0%	
		Environmental samples	Soil samples		58.9%	
			Lairage swabs		65.8%	
			Animal feed		30.4%	
			Water		18.8%	
2023	Spain	Cow	Feces	*Listeria ivanovii*	0.5%	[71]
			Tonsils		1.1%	
			Udder		7.1%	
2024	Costa Rica, USA	Chicken	chicken meat	*Salmonella enterica*	58.5%	[72]
			chicken caecal		38.0%	
2020	Argentina	Poultry meat supply chain	Poultry	Thermotolerant *Campylobacter*	33%	[73]
			Wild-living birds		24%	
			Darkling beetles		20%	
			Farm workers boots		17%	
			Darkling beetle larvae		10%	
			Flies		5%	
			Litter		5%	

## Data Availability

No new data were created or analyzed in this study. Data sharing is not applicable to this article.
